# Solamargine, a bioactive steroidal alkaloid isolated from *Solanum aculeastrum* induces non-selective cytotoxicity and P-glycoprotein inhibition

**DOI:** 10.1186/s12906-018-2208-7

**Published:** 2018-05-02

**Authors:** Trevor Burger, Tsholofelo Mokoka, Gerda Fouché, Paul Steenkamp, Vanessa Steenkamp, Werner Cordier

**Affiliations:** 1 Department of Pharmacology, School of Medicine, Faculty of Health Sciences, University of Pretoria, P.O.Box X323, Pretoria, Arcadia 0007 South Africa; 2 Biosciences Division, Council of Scientific and Industrial Research, Pretoria, South Africa; 3 Department of Biochemistry, University of Johannesburg, Auckland Park, Johannesburg, South Africa

**Keywords:** Cancer, P-glycoprotein, *Solanum aculeastrum*, Solamargine, Solasonine, Steroidal alkaloids

## Abstract

**Background:**

*Solanum aculeastrum* fruits are used by some cancer sufferers as a form of alternative treatment. Scientific literature is scarce concerning its anticancer activity, and thus the aim of the study was to assess the in vitro anticancer and P-glycoprotein inhibitory potential of extracts of *S. aculeastrum* fruits. Furthermore, assessment of the combinational effect with doxorubicin was also done.

**Methods:**

The crude extract was prepared by ultrasonic maceration. Liquid-liquid extraction yielded one aqueous and two organic fractions. Bioactive constituents were isolated from the aqueous fraction by means of column chromatography, solid phase extraction and preparative thin-layer chromatography. Confirmation of bioactive constituent identity was done by nuclear magnetic resonance and ultra-performance liquid chromatography mass spectrometry. The crude extract and fractions were assessed for cytotoxicity and P-glycoprotein inhibition in both cancerous and non-cancerous cell lines using the sulforhodamine B and rhodamine-123 assays, respectively.

**Results:**

Both the crude extract and aqueous fraction was cytotoxic to all cell lines, with the SH-SY5Y neuroblastoma cell line being most susceptible to exposure (IC_50_ = 10.72 μg/mL [crude], 17.21 μg/mL [aqueous]). Dose-dependent P-glycoprotein inhibition was observed for the crude extract (5.9 to 18.9-fold at 100 μg/mL) and aqueous fraction (2.9 to 21.2 at 100 μg/mL). The steroidal alkaloids solamargine and solanine were identified. While solanine was not bioactive, solamargine displayed an IC_50_ of 15.62 μg/mL, and 9.1-fold P-glycoprotein inhibition at 100 μg/mL against the SH-SY5Y cell line. Additive effects were noted for combinations of doxorubicin against the SH-SY5Y cell line.

**Conclusions:**

The crude extract and aqueous fraction displayed potent non-selective cytotoxicity and noteworthy P-glycoprotein inhibition. These effects were attributed to solamargine. P-glycoprotein inhibitory activity was only present at concentrations higher than those inducing cytotoxicity, and thus does not appear to be the likely mechanism for the enhancement of doxorubicin’s cytotoxicity. Preliminary results suggest that non-selective cytotoxicity may hinder drug development, however, further assessment of the mode of cell death is necessary to determine the route forward.

## Background

Cancer is a prominent contributor to worldwide mortality [1]. According to the World Health Organisation, 14.1 million new cases of cancer were diagnosed in 2012, with 8.2 million cancer-related deaths reported between 2008 and 2012 [2]. In South Africa, more than 100,000 people are diagnosed with cancer annually, with prostate and breast cancer as the most frequently found [2].

Anticancer therapy includes options such as surgical interventions, radiation therapy and chemotherapy [1]. Due to cost, adverse effects [3, 4] and increasing resistance, the above mentioned therapeutic options are often subject to treatment failure [5]. Contributing resistance mechanisms include, among others, the over-expression of efflux transporters in cancer cells which aid in removing cytotoxins from the intracellular compartment [6]. The P-glycoprotein (P-gp) transporter has been implicated in such resistant cancer phenotypes [7]. P-gp is a 170 kDa ATP-binding cassette membrane transporter which is expressed throughout the body [8]. Up-regulation of P-gp is associated with the majority of drug-resistant cancers, as it effluxes anticancer molecules such as doxorubicin, thus hindering its efficacy [7, 9].

Combination therapy has been suggested to counteract resistance, which allows for drugs to be used together at lower concentrations. This in turn may reduce side effects and potentially overcome resistance [10]. Doxorubicin is used alone or in combination to treat several cancers, solid tumours and soft tissue sarcomas [11]. However, with the increased frequency of cancer and lack of clinical effectivity of many anticancer drugs, novel treatments are sought [12]. Herbal remedies provide an ideal source of such chemical entities.

Complementary and alternative medicines are used worldwide for treatment of various diseases [13]. Numerous anticancer agents have been isolated from plants, or are derivatives thereof: vinblastine and vincristine (*Catharanthus roseus);* taxanes (*Taxus canadensis*); betulinic acid (*Betula* spp.), etoposide (*Podophyllum* spp.) and combretastatin (*Combretum caffrum)* [14–16]. Africa is a rich source of potentially bioactive phytochemicals, giving credence to the investigation of such herbal remedies against cancer.

The fruits of *Solanum aculeastrum* Dunal. (family Solanaceae), also known as the goat apple, are sometimes consumed by cancer patients as they believe it will be beneficial to treatment [17]. Literature concerning the anticancer activity of extracts is scarce, however, steroidal alkaloids such as solamargine have been shown to possess such properties [18, 19]. The aim of the study was to assess the in vitro anticancer activity of alkaloid-enriched fractions from *S. aculeastrum* fruits by determining its cytotoxicity, P-gp inhibition, and effect in combination with doxorubicin. Furthermore, the bioactive constituents was isolated and identified.

## Methods

### Plant material

Ripened fruits of *S. aculeastrum* were gifted from the Makana Botanical Gardens in Grahamstown (Eastern Cape, South Africa), where the identity was confirmed by Ms. Karin Cockburn. Specimens are available within the botanical gardens, while plant material is stored within the Department of Pharmacology as sectioned and powdered plant material. Fruits were cut and air-dried at ambient temperature. Plant material was ground to a fine powder (Yellowline A10, Merck (Pty) Ltd) and stored in air-tight, amber containers until needed.

### Preparation of crude extract

Ground plant material (150 g) was sonicated in 1.5 L methanol for 30 min, after which it was agitated for 2 h on a shaker. The solution was incubated for an additional 16 h at 4 °C. The supernatant was collected, and the marc re-extracted five more times. Supernatants were pooled, centrifuged at 500 *g* for 1 min, vacuum-filtered (0.2 μm filters, Waters Corporation) and concentrated using in vacuo rotary evaporation (Büchi Rotovapor R-200, Büchi). Dried crystals were resuspended in distilled water (dH_2_O) and lyophilized (Freezone® 6 Freeze Dry System, Labconco) to yield a dry, yellow powder (17.98% *w*/w).

### Preparation of alkaloid-enriched fractions

Alkaloid-enriched fractions were prepared as described by Munari et al. [20] with modifications to the volumes used. Two organic fractions were obtained by sequential liquid-liquid extraction with diethyl ether and chloroform. The crude extract (26.72 g) was acidified with 2% acetic acid (267.2 mL) on a shaker for 2 h. Diethyl ether (534.4 mL) was added to the acidified mixture, shaken for 20 min and the organic phase siphoned off after separation. This procedure was repeated four times, and all diethyl ether fractions combined. After fractionation, the aqueous phase was fractionated with chloroform as described above. Organic fractions were clarified with anhydrous sodium sulphate (10% *w*/*v*). The two organic fractions (diethyl ether [8.98% *w*/w] and chloroform [0.15% w/w]) and the aqueous alkaloid-enriched fraction were concentrated using in vacuo rotary evaporation and lyophilisation, respectively. All samples were dissolved in dimethyl sulfoxide (DMSO). Aliquots (20 mg/mL) were stored at -80 °C until needed.

### Test for the presence of steroidal alkaloids

Samples (20 μg) were spotted onto a C10 silica plate (5 × 10 cm, Agilent Technologies South Africa) and developed in a mobile phase consisting of chloroform, acetone and methanol (4:4:2). Plates were visualized using ultraviolet light (UV, at 254 and 366 nm), sprayed with Dragendorff’s reagent and developed in an oven at 60 °C.

### Cellular assays

#### Cell maintenance

The Caco-2, DU145, HepG2, MCF-7, MDA-MB-231, SK-Br3, 3 T3-L1, C2C12 and SC-1 cell lines were obtained from the American Tissue Culture Collection (ATCC), while the A2780 cell line was obtained from the European Collection of Authenticated Cell Cultures. The SH-SY5Y cell line was gifted from North-West University’s Department of Pharmacology, which was originally purchased from the ATCC. The Ea.hy926 cell line was gifted by Dr. C Edgell.

Cells were grown in either Dulbecco’s Modified Eagle Medium (DMEM; 3 T3-L1 pre-adipocytes, C2C12 myoblast, EA.hy926 hybrid endothelial, MCF-7 breast carcinoma, MDA-MB-231 breast adenocarcinoma cell lines), Eagle’s Minimum Essential Medium (EMEM; Caco-2 colon carcinoma, HepG2 hepatocarcinoma, SC-1 mouse fibroblast cell lines), Hams F12 Nutrient Medium (Hams-F12, SH-SY5Y neuroblastoma) or Roswell Park Memorial Institute-1640 (RPMI-1640; A2780 ovarian carcinoma, DU145 prostate carcinoma, Sk-Br3 breast adenocarcinoma) supplemented with 10% foetal calf serum (FCS) and 1% penicillin/streptomycin at 37 °C in a humidified atmosphere of 5% CO_2_. Adherent cells were harvested through trypsination and centrifugation at 200 *g* for 5 min. Cells were counted using the trypan blue exclusion assay and resuspended at the desired concentration (HepG2 = 2 × 10^5^ cells/mL; 3 T3-L1, A2780, C2C12, Caco-2, DU145, EA.hy926, MCF-7, MDA-MB-231, SC-1, Sk-BR-3 and SH-SY5Y = 1 × 10^5^ cells/mL). Cells (100 μL) were seeded into clear 96-well plates and allowed to attach overnight.

#### Cytotoxicity evaluation

The effect of the crude extract and fractions on cell density was determined using the sulforhodamine B (SRB) colourimetric assay as described by Vichai and Kirtikura [21], with minor modifications. Cells were exposed to 100 μL FCS-free medium (negative control [NC]), DMSO (0.5%, vehicle control [VC]), saponin (1%, positive control [PC]) or sample (2, 6.4, 20, 64 and 200 μg/mL) for 24 or 72 h. Cells were fixed overnight at 4 °C with 50 μL trichloroacetic acid (50%). Plates were washed four times with tap water and allowed to dry at 40 °C in an oven. Fixed protein elements were stained with 100 μL SRB (0.057% in 1% acetic acid) and incubated for 30 min. Plates were washed with 100 μL acetic acid (1%) thrice and allowed to dry. Bound dye was dissolved with 200 μL Tris-base solution (10 mM, pH 10.5). Absorbance was measured at 510 nm (reference 630 nm) using an ELX800UV plate reader (Bio-Tek Instruments, Inc.). Four cell lines were selected for the remainder of the study based on the cytotoxicity evaluation: the two most susceptible cancerous cell lines (SH-SY5Y and SK-Br3), as well as one susceptible (EA.hy926) and one non-susceptible (C2C12) non-cancerous cell line.

#### P-glycoprotein inhibition

The rhodamine-123 accumulation assay was used to assess P-gp inhibitory activity according to Jia and Wasan [22] with modifications to volumes and incubation times. Cells were seeded as described in Section 2.5.1. and allowed to attach for 48 h in white 96-well plates. Wells were exposed to 100 μL phosphate-buffered saline (PBS) (blank and NC), DMSO (VC, 0.5%), samples (2, 6.4, 20, 64 and 200 μg/mL) or verapamil (PC; 2, 6.4 and 20 μM) prepared in PBS, and incubated for 1 h at 37 °C. After incubation, 40 μL rhodamine-123 (10 μM) was added for 1 h. Cells were washed twice with PBS and resuspended in 100 μL PBS. Fluorescence was measured using a FLUOstar OPTIMA plate reader (BMG Labtech) at 485 nm (excitation) and 520 nm (emission). After measurement, cell density was assessed using the SRB assay to normalise data to avoid misinterpretation due to altered cell density. Data was blank-excluded, fluorescence intensity normalised to cell density and expressed as a fold change relative to the negative control.

### Isolation of active compound

#### Bioassay-guided fractionation

The most active fraction (aqueous alkaloid-enriched fraction) was subjected to high performance liquid chromatography (HPLC; Agilent 1200 HPLC System, Agilent Technologies South Africa) using acetonitrile and dH_2_O (gradient: 0–10% acetonitrile between 0 and 5 min, to 100% at 30 min, total run time: 35 min) and a Sunfire C18 semi-preparative column (150 mm × 10 mm, particle size: 10 μm). Samples (25 mg/mL) were repeatedly injected and fractionated into eleven sub-fractions collected every 2 min. Each sub-fraction was reconstituted to the desired concentration in DMSO. Cytotoxicity and P-gp inhibition was assessed against the SK-Br3 breast carcinoma cell line at 50 μg/mL. Sub-fractions 10 and 11 were most active, and thus further assessed.

#### Isolation of active constituents by column and solid phase extraction chromatography

Silica gel was mixed with chloroform and methanol (3:2) and poured into a cotton wool plugged glass column (2.7 × 50.5 cm). The aqueous alkaloid-enriched fraction was dissolved in a hydromethanolic solution (10% dH_2_O), mixed with silica, left at room temperature to dry and loaded on top of the packed silica gel column. Sub-fractions were collected in glass tubes, monitored using TLC, and compared to sub-fractions 10 and 11 as reference. Similar sub-fractions were pooled together and further purified by solid phase extraction chromatography using a ISOLUTE flash C18 column with a mobile phase of acetonitrile and dH_2_O (starting at 100% dH_2_O, followed by 5% acetonitrile, then 10% acetonitrile and increasing to 100% acetonitrile in 10% increments). Major compounds co-eluted at 40% acetonitrile as a white-powder and were separated using preparative TLC (solvent system: methanol, ethyl acetate and acetone; 4:4:2) to afford compound 1 and compound 2.

The identities of the isolated compounds were confirmed using Nuclear Magnetic Resonance (NMR, 600 MHz VNMRS, Varian) and Ultra-Performance Liquid Chromatography Tandem Mass-Spectrometry (Synapt G1 UPLC-QTOF-HDMS system, Waters, USA) analysis. Compounds were analysed using different NMR techniques such as H-1-NMR, C-13-NMR, Heteronuclear Single Quantum Coherence (HSQC), Heteronuclear Multiple Bond Correlation (HMBC) and correlation spectroscopy (COSY) to accurately determine structural moieties. Mass Lynx 4.1 software was used for analysis of mass spectrometry data and the fragmentation patterns of the isolated compounds were identified using Waters MassFragment software (version 2.0.w.15).

#### UPLC-TOF-MS fingerprinting of the crude extract and alkaloid-enriched fractions

Samples (20 mg/mL) were subjected to UPLC-QTOF-HDMS analysis in order to screen for major constituents. The relative abundance of the major compounds was compared between samples by equalising the intensity scale between chromatograms of different samples. The highest sample intensity was used as the scale standard for other chromatograms.

#### Bioactivity

Compounds 1 and 2 (0.32, 1, 3.2, 32 and 50 μg/mL) were assessed for cytotoxicity against the C2C12, EA.hy29, SH-SY5Y and SK-Br3 cell lines after 72 h exposure. P-gp inhibitory activity was assessed in the SH-SY5Y and EA.hy926 cell lines only. Only the most active compound was subjected to synergistic investigation with doxorubicin.

### Synergistic potential evaluation of the aqueous alkaloid-enriched fraction and active isolated compound in combination with doxorubicin

The synergistic potential of the aqueous alkaloid-enriched fraction and active compound in combination with doxorubicin was assessed using the method of Kars et al. [23]. The half maximal inhibitory concentration (IC_50_) of the aqueous alkaloid-enriched fraction was reassessed, as well as the cytotoxicity of doxorubicin against the C2C12, EA.hy29, SH-SY5Y and SK-Br3 cell lines. The active isolated compound, aqueous alkaloid-enriched fraction and doxorubicin was tested at two-, one-, half- and a quarter-fold the respective IC_50_ values in a checkerboard fashion.

Effects of the combination between doxorubicin and the extract/fractions were presented as a fractional inhibitory index (FIX):

$$ FIX= FIC(A)+ FIC(B) $$

A FIX value < 0.5 is indicative of synergism, between 0.5–1 as additive effect, between 1 and 2 as an indifferent effect and > 2 as antagonism.

### Statistics

All experiments were performed with technical and biological triplicates. Results were presented as the mean ± SEM. Statistical analyses were done using GraphPad Prism 5.0. Non-linear regression was used to determine the IC_50_. Kruskal-Wallis analysis with a post-hoc Dunn’s test was used to compare controls to samples. Significance was taken as *p* < 0.05.

## Results

### Isolation and structural elucidation of steroidal alkaloids

Intense black and violet spots were observed for the crude extract and alkaloid-enriched fraction on TLC plates under short (254 nm) and long (366 nm)-wavelength UV light, respectively. Orange spots were seen after spraying with Dragendorff’s reagent. The aqueous alkaloid-enriched fraction was sub-fractionated into eleven fractions. Two major compounds were identified in sub-fractions 10 and 11, which were not visible under UV light, but appeared after vanillin-spraying.

**Fig. 1:**
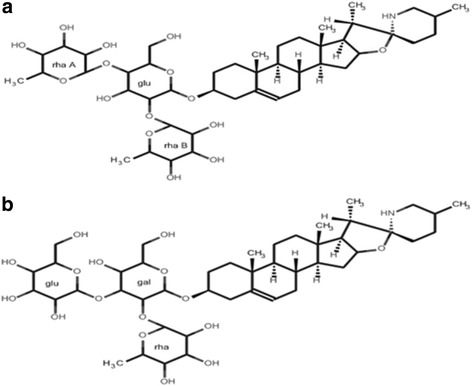
Chemical structure of solamargine (**a**) and solasonine (**b**). gal: galactose; glu: glucose; rha: rhamnose

Compound 1 appeared as white crystals. The structure of compound 1 was identified by H-1-NMR, C-13-NMR, 2-D data analysis. C-13-NMR revealed that compound 1 possesses an aglycone backbone related to a steroidal spirazolane-type alkaloid. Four quaternary carbons at chemical shifts (δ_c_’s) 38.2, δ_c_ 41.8 ppm including one linked to oxygen and nitrogen at δ_c_ 99.6 as well as one attached to a double bond at δ_c_ 142.1, nine methine groups at δ_c_’s 31.8, 31.8, 42.9, 51.9, 57.9, 64.2, 79.5, 80.5, 122.8, ten methylene groups at δ_c_’s 22.2, 30.9, 31.1, 32.9, 33.1, 33.4, 38.7, 39.7, 41.2, 48.5 ppm and four methyl groups at δ_c_’s 15.6, 17.0, 19.9, 19.9 ppm were observed (Table 1). An ether function with a trisaccharide moiety showing an anomeric carbon at δ_c_ 100.6 linked to the oxygen of C-3 at δ_c_ 79.5 was also present (Table 1). The 1D NMR chemical shifts of the trisaccharide moiety indicated the structure *O*-[α-L-rhamnopyranosyl-(1 → 2)-*O*-[α-L-rhamnopyranosyl-(1 → 4)]-β-D-glucopyranoside. The proton NMR showed four distinctive aglycone methyls at δ_c_ 0.83 (3H, s), 0.85 (3H, d, *J* [coupling constant] 6.4), 0.97 (3H, d, *J* 7.2) and 1.05 (3H, s) (Table 1). Two multiplets observed at δ_c_ 1.26 were attributable to 6-deoxyhexose methyls whereas a doublet at δ_c_ 5.38 with *J* value of 4.6 could be attributed to an olefinic proton at position C-6. Four anomeric H-1-NMR resonances were observed at δ_c_ 4.34 (1H, m), 4.50 (1H, d, *J* 7.6), 4.84 (1H, s) and 5.21 (1H, s) (Table 1). High resolution mass spectrometry (HRMS) data for compound 1 yielded the molecular formula C_45_H_73_NO_15_ with a molecular mass of *m/z* (mass to charge ratio) 868.5077 ([M + H]^+^, 100%) which was identified as the steroidal alkaloid, solamargine (Fig. 1).

**Table 1:** H-1-NMR and C-13-NMR resonances of compound 1 compared to solamargine

		Solamargine (^19^) (deuterated chloroform and methanol)		Compound 1 (2015) (deuterated methanol)	
	Carbon no (type)	C13-NMR	H-1-NMR (J in Hz)	C13-NMR	H-1-NMR (J in Hz)
Aglycone	C-1 (CH_2_)	37.9		38.70	
	C-2 (CH_2_)	30.5		31.13	
	C-3 (CH)	79		79.50	
	C-4 (CH_2_)	40.4		41.19	
	C-5 (C)	141.1		142.07	
	C-6 (CH)	122.2	5.37 (d, J 4.2)	122.78	5.38 (d, J 4.6)
	C-7 (CH_2_)	32.5		32.94	
	C-8 (CH)	32.1		31.81	
	C-9 (CH)	50.7		51.90	
	C-10 (C)	37.5		38.20	
	C-11 (CH_2_)	21.4		22.15	
	C-12 (CH_2_)	38.9		39.67	
	C-13 (C)	41.1		41.77	
	C-14 (CH)	57.1		57.90	
	C-15 (CH_2_)	30.1		30.90	
	C-16 (CH)	79.7	4.33 (m)	80.45	4.34 (m)
	C-17 (CH)	63.2		64.21	
	C-18 (CH_3_)	16.8	0.84 (s)	17.00	0.83 (s)
	C-19 (CH_3_)	19.6	1.05 (s)	19.91	1.05 (s)
	C-20 (CH)	42		42.85	
	C-21 (CH_3_)	15.4	0.97 (d, J 7.0)	15.60	0.97 (d, J 7.2)
	C-22 (C)	98.9		99.60	
	C-23 (CH_2_)	34.4		33.36	
	C-24 (CH_2_)	32.7		33.14	
	C-25 (CH)	31.3		31.81	
	C-26 (CH_2_)	47.8	2.6 (m, br)	48.47	2.53 (m)
	C-27 (CH_3_)	19.6	0.87 (d, J 6.0)	19.98	0.85 (d, J 6.4)
Glucose (glu)	C-1′ (CH)	99.9	4.48 (d, J 7.8)	100.63	4.50 (d, J 7.6)
	C-2′ (CH)	79.5		80.23	
	C-3’(CH)	77.3		78.19	
	C-4′ (CH)	75.7		76.73	
	C-5′ (CH)	78.6		79.50	
	C-6′ (CH_2_)	61.4		62.12	
Rhamnose A (rha A)	C-1″ (CH)	102.4	4.87 (s)	103.16	4.84 (s)
	C-2″ (CH)	71.8		72.60	
	C-3″ (CH)	71.5		72.35	
	C-4″ (CH)	73.3		74.10	
	C-5″ (CH)	69.1		69.94	
	C-6″ (CH_3_)	17.7	1.29 (m)	18.11	1.26 (m)
Rhamnose B (rha B)	C-1″‘(CH)	101.5	5.23 (s)	102.44	5.21 (s)
	C-2″‘(CH)	71.6		72.52	
	C-3″‘(CH)	71.3		72.35	
	C-4″‘(CH)	73		73.89	
	C-5″‘(CH)	70.1		70.85	
	C-6″‘(CH_3_)	17.6	1.29 (m)	18.0.	1.26 (m)

Extensive evaluation of the similarities in characteristic signals of the H-1-NMR, C-13-NMR and 2-D NMR for compound 2 revealed that it was like compound 1. The structure included an aglycone backbone attached to a trisaccharide moiety. However, differences were present in the trisaccharide sugar moiety in which a O-[α-L-rhamnopyranosyl-(1 → 2)-O-[b-glucopyranosyl-(1 → 3)]-β-D-galactopyranoside structure was observed. HRMS data for compound 2 yielded the molecular formula C_45_H_73_NO_16_ with molecular weight peak at *m/z* 884.5205 ([M + H]^+^, 100%) which was identified as the steroidal alkaloid solasonine (Fig. 1). The mass difference between solasonine and solamargine is consistent with the additional hydroxyl group seen in solasonine’s structure.

UPLC-QTOF-HDMS analysis of the crude extract and alkaloid-enriched fractions revealed that solamargine and solasonine with *m/z’s* of 868.4987 and 884.4989 ([M + H]^+^, 100%), respectively, were the two major constituents. Although there were similarities in composition between samples, the concentration of major compounds differed. The crude extract and aqueous alkaloid-enriched fraction had a significantly higher abundance of solamargine and solasonine when compared to the chloroform and ether fractions.

### Cytotoxicity

Samples were cytotoxic in cancerous (A2780, Caco-2, DU-145, HepG2, MCF-7, MDA-MB-231, SH-SY5Y and SK-Br3) and non-cancerous (3 T3-L1, C2C12, EA.hy.926 and SC-1) cell lines (Table 2). The crude extract and aqueous alkaloid-enriched fraction displayed noteworthy (< 30 μg/mL) and moderate-to-low cytotoxicity (> 30 μg/mL) after 72 h exposure (Table 2). In most cell lines, cytotoxicity was evident as early as 24 h exposure, which either plateaued or decreased (1-to-2-fold increase) after 72 h exposure. Variable cytotoxicity towards non-cancerous cell lines was observed, with IC_50_ values from 3.88 to 93.41 μg/mL and 2.79 to 91.18 μg/mL after 24 h and 72 h exposure, respectively. The chloroform and diethyl ether fractions had negligible cytotoxicity. The aqueous alkaloid-enriched fraction had a potent dose-dependent effect on the SH-SY5Y (IC_50_ = 17.21 μg/mL), Sk-Br3 (IC_50_ = 18.81 μg/mL) and HepG2 (IC_50_ = 20.66 μg/mL, Table 2) cell lines, but displayed low cytotoxicity in the C2C12 (IC_50_ = 62.00 μg/mL) cell line after 72 h exposure. The EA.hy926 cell line was most susceptible to the aqueous alkaloid-enriched fraction with an IC_50_ of 9.35 μg/mL after 72 h, with a narrow cytotoxic range.

**Table 2:** Cytotoxicity of the crude extract, aqueous alkaloid-enriched fraction and solamargine as described by their IC_50_ values in a panel of cancerous and non-cancerous cell lines

	IC_50_ ± SEM											
	Cancerous cell lines								Non-cancerous cell lines			
Extract/fraction^a^	A2780	Caco-2	DU-145	HepG2	MCF-7	MDA-MB-231	SH-SY5Y	SK-Br3	3 T3-L1	C2C12	EA.hy.926	SC-1
Crude 24 h^a^	34.73 ± 1.07	36.46 ± 1.20	74.33 ± 1.14	9.44 ± 1.11	44.35 ± 1.06	26.87*	19.10 ± 1.07	33.25 ± 1.10	37.24 ± 1.34	93.41 ± 1.08	3.88 ± 1.07	28.73 ± 1.09
Crude 72 h^a^	32.88*	24.40 ± 1.13	47.11 ± 1.18	7.04 ± 1.08	27.94 ± 1.11	24.00*	12.97 ± 1.07	20.11 ± 1.08	19.55 ± 1.23	91.18 ± 1.13	2.79 ± 1.55	23.07 ± 1.10
Aqueous 24 h^a^	31.45*	50.37 ± 1.26	46.53 ± 1.21	40.02 ± 1.08	31.91 ± 1.06	28.05*	29.11 ± *	29.24*	21.95 ± 1.50	> 100	16.45 ± 1.06	19.89 ± 1.09
Aqueous 72 h^a^	29.81*	25.21 ± 1.12	34.08*	20.66 ± 1.10	25.49 ± 1.13	26.11*	17.21 ± 1.15	18.81 ± 1.14	12.89 ± 1.23	62.00 ± 1.22	9.35 ± 1.08	21.10 ± 1.08
Solamargine 72 h^b^	X	X	X	X	X	X	15.62 ± 1.45	18.59 ± 1.13	X	20.25 ± 1.08	8.30 ± 1.12	X
P-gp inhibitor												
Verapamil 24 h^b^	93.48 ± 1.05	> 100	> 100	18.72 ± 1.08	> 100	> 100	> 100	> 100	> 100	> 100	31.55 ± 1.12	> 100
Verapamil 72 h^b^	65.75 ± 1.06	> 100	> 100	8.34 ± 1.08	46.76 ± 1.14	47.5 ± 1.09	> 100	41.92 ± 1.09	79.22 ± 1.21	> 100	5.69 ± 1.11	60.01 ± 1.16

* Ambiguity in data points (GraphPad 5.0 software could not predict SEM due to steepness of dose-response curve)

^a^presented in μg/mL

^B^presented in μM

^X^Not tested on mentioned cell line

Sub-fractions 10 and 11 from the aqueous alkaloid-enriched fraction displayed potent cytotoxicity (~ 91%) at 50 μg/mL in the SK-Br3 cell line. Bioactivity-guided fractionation and isolation procedures identified solamargine as the bioactive constituent. Solamargine induced dose-dependent cytotoxicity in the SH-SY5Y and SK-Br3 cell lines with IC_50_ values of 15.62 μM (13.54 μg/mL) and 18.59 μM (16.12 μg/mL) after 72 h incubation, respectively (Table 2). Solasonine was not cytotoxic (IC_50_ > 56.63 μM or 50 μg/mL).

### P-glycoprotein inhibition

**Fig. 2:**
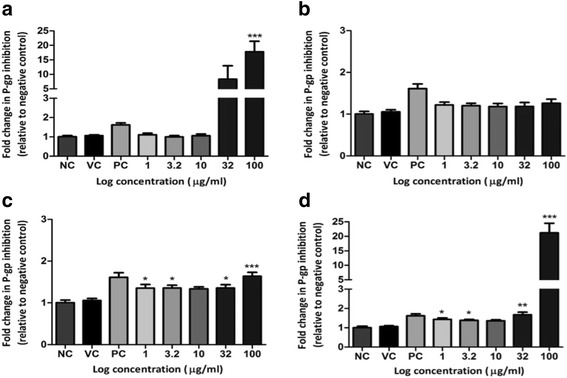
P-gp inhibitory potential in the SH-SY5Y neuroblastoma cell line when exposed to the **a**) crude extract, **b**) diethyl ether fraction, **c**) chloroform fraction and **d**) aqueous alkaloid-enriched fraction. PC: verapamil (1 μM). * *p* < 0.05*,* ** *p* < 0.01, *** *p* < 0.001

The crude extract and the aqueous alkaloid-enriched fraction induced dose-dependent P-gp inhibition (2.87 to 21.2-fold) at 100 μg/mL (Fig. 2A and D). The chloroform and diethyl ether fraction displayed poor P-gp inhibition (1.12 to 1.63-fold). The diethyl ether, chloroform and aqueous alkaloid-enriched fractions showed the greatest inhibitory potential towards the SH-SY5Y cell line (Fig. 2B to 2D) with inhibition of 1.26, 1.64 and 21.21-fold at 100 μg/mL, respectively. The fractions exhibited the lowest activity towards the C2C12 cell line with inhibitory values of 1.13, 1.27 and 2.87-fold at 100 μg/mL, respectively. The aqueous alkaloid-enriched fraction displayed inhibitory activity across all cell lines, with values of 2.82 to 21.21-fold at 100 μg/mL (*p* < 0.001). The P-gp inhibition exhibited by the crude extract and aqueous alkaloid-enriched fraction was similar (5.89 to 18.88-fold at 100 μg/mL [*p* < 0.001]). Significant (*p* < 0.05 and 0.001) but low P-gp inhibition was induced by the chloroform fraction in the SH-SY5Y cancerous cell line (Fig. 2C) with an inhibitory value of 1.64-fold at 100 μg/mL. The diethyl ether fraction displayed low but significant (*p* < 0.01) inhibition in the EA.hy926 cell line of 1.47-fold at 100 μg/mL. No significant activity was noted for any other cell line tested.

**Fig. 3:**
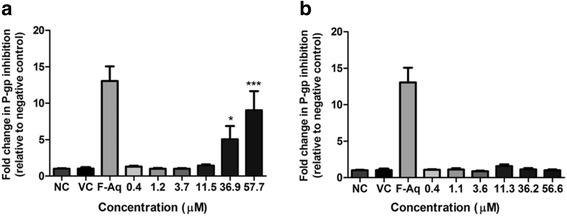
P-gp inhibitory potential of solamargine (**a**) and solasonine (**b**) against the SH-SY5Y neuroblastoma cell line. * *p* < 0.05, *** *p* < 0.001

Solamargine caused significant (*p* < 0.05 and *p* < 0.001) dose-dependent P-gp inhibition in the SH-SY5Y cancerous cell line (Fig. 3A) with 5.07-fold activity at 36.9 μM (32 μg/mL) and 9.10-fold activity at 57.7 μM (50 μg/mL). However, the effect was 3.95-fold less when compared to the aqueous alkaloid-enriched fraction (13.05-fold) at 50 μg/mL (Fig. 3A). Solamargine inhibited P-gp more in the EA.hy926 (13.81-fold) non-cancerous cell line when compared to the SH-SY5Y (9.05-fold) cancerous cell line at 57.70 μM (50 μg/mL). Solasonine did not inhibit P-gp activity (Fig. 3B).

### Synergistic potential of the aqueous alkaloid-enriched fraction and solamargine with doxorubicin

A dose-dependent decrease in cell density was observed after exposure to doxorubicin, with more selectivity towards cancerous cell lines (Table 3). SH-SY5Y cells were most susceptible to doxorubicin (IC_50_ of 56.60 nM). No synergistic effects were observed between doxorubicin and the aqueous alkaloid-enriched fraction or solamargine (Table 3). Additive effects were observed in the SH-SY5Y cell line for the doxorubicin combinations with the aqueous alkaloid-enriched fraction (FIX value = 0.71) and solamargine (FIX value = 0.51). Additive effects were also observed in the SK-Br3 and EA.hy926 cell lines when exposed to the combination with solamargine (FIX value = 0.66) and the aqueous alkaloid-enriched fraction (FIX value = 0.94), respectively. In contrast, antagonistic effects were observed in the C2C12 cell line when exposed to combinations with the aqueous alkaloid-enriched fraction (FIX value = 2.10) and solamargine (FIX value = 2.53). Indifferent effects were noted when the SK-Br-3 and EA.hy926 cell lines were exposed to the combination with the aqueous alkaloid-enriched fraction and solamargine, respectively (Table 3, FIX values: > 1, however < 2).

**Table 3:** Synergistic potential of solamargine and the aqueous alkaloid-enriched fraction as described by their FIX values in a panel of cancerous and non-cancerous cell lines

Cell type	Cancerous				Non-cancerous			
Cell line	SH-SY5Y		SK-Br3		C2C12		EA.hy926	
Compound/Fraction	Solamargine	Aqueous alkaloid-enriched fraction	Solamargine	Aqueous alkaloid-enriched fraction	Solamargine	Aqueous alkaloid-enriched fraction	Solamargine	Aqueous alkaloid-enriched fraction
IC_50_ of (A) [IC_50_ sample + doxorubicin (μM or μg/mL)]	3.4	7.39	4.3	11.18	30.77	15.7	5.5	2.62
IC_50_ of (B) [IC_50_ doxorubicin + sample (nM)]	16.27	24.22	40.83	47.48	149.9	140.7	213.4	220
IC_50_ (A) [Solamargine (μM) or aqueous alone (μg/mL)]	15.62	26.5	18.59	19.7	20.25	13.7	8.3	7.3
IC_50_ (B) [doxorubicin alone (nM)]	56.6	56.6	94.1	94.1	148.5	148.5	381.6	381.6
FIC (A)	0.22	0.28	0.23	0.57	1.52	1.15	0.66	0.36
FIC (B)	0.29	0.43	0.43	0.5	1.01	0.95	0.56	0.58
FIX value	0.51	0.71	0.66	1.07	2.53	2.1	1.22	0.94
Result	Additive effect	Additive effect	Additive effect	Indifferent effect	Antagonistic effect	Antagonistic effect	Indifferent effect	Additive effect

## Discussion

Steroidal alkaloids were positively identified in the crude extract and alkaloid-enriched fractions using both UV light and Dragendorff’s reagent. Dragendorff’s reagent is known to produce orange spots when reacting with alkaloids [24, 25]. Literature supports the presence of such alkaloids in *S. aculeastrum* [26]. The two most prominent steroidal alkaloids, solamargine (compound 1) and solasonine (compound 2), were isolated. Structural analysis was found to compare well with literature [19, 27]. According to literature, more than 100 *Solanum* spp., including *S. aculeastrum*, contain solamargine and solasonine [28]. Other compounds isolated from *S. aculeastrum* include the steroidal alkaloids, solaculine A, solasodine and tomatidine [19, 26]. Solanopubamine and solanidine have also been noted in other *Solanum* spp. [29, 30].

Both the crude extract and aqueous alkaloid-enriched fraction were highly cytotoxic in the panel of cell lines. A mixture of concentration- and time-dependent cytotoxicity was observed. A few isolated studies have assessed the cytotoxicity of *S. aculeastrum*, but not as broad as in the present study. Koduru et al. [17] reported that a methanol fruit extract displayed a narrow cytotoxic range, with an IC_50_ of 17.8 μg/mL in the MCF-7 cell line [17]. Although the present study displayed greater cytotoxicity (IC_50_ = 10.14 μg/mL), a narrow cytotoxic range was also observed. Other *Solanum* species, such as *S. nigrum* and *S. incanum*, have also been shown to be cytotoxic [31, 32]. *S. incanum* has been shown to induce both an antiproliferative and cytotoxic effect via cell cycle arrest and apoptosis [32].

The cytotoxicity observed for solamargine in the present study is similar to that described in literature (between 5.28 to 21.03 μM [4.58 to 18.23 μg/mL]) [20]. Solamargine alters cell morphology, induce chromatin condensation, and fragment DNA in hepatoma (Hep3B) cells, suggesting a pro-apoptotic effect [33]. Apoptosis appears to occur via intrinsic and extrinsic apoptotic pathways in breast cancer cells [34]. Furthermore, solamargine induces extracellular signal-regulated protein kinases 1 and 2 (ERK1/2) with downstream cytotoxicity in human lung cancer [35]. Solamargine may also deter metastatic effects in cancer by reducing its invasive and migratory potential [36]. In the present study, solasonine was not cytotoxic. Conflicting results have been reported, which either support [37] or contradict [20] the current findings. Conformational changes in solasonine’s molecular structure could account for the differences in cytotoxicity [38].

The non-selective cytotoxicity observed for the samples render the extracts and solamargine as poor candidates for further drug development, unless structural alterations could improve its selectivity for cancerous cell lines. Similar results were reported by Munari et al. [20], where an ethanol extract from *S. lycocarpum* fruits, as well as solamargine, displayed antiproliferative effects towards human and Chinese hamster lung fibroblast cell lines [20].

The crude extract and aqueous alkaloid-enriched fraction displayed prominent P-gp inhibitory activity, which could be ascribed to the presence of solamargine, however, other phytochemcials cannot be excluded. The P-gp inhibitory activity was only seen at concentrations greater than that inducing cytotoxic effects, and also displayed non-selective inhibition. Literature is scarce regarding P-gp inhibitory activity of *Solanum* spp. An ethanol extract from *S. trilobatum* was found to induce significant (*p* < 0.05) inhibition when compared to verapamil, however, only at 300 μg/mL [39]. This species also contains solamargine and solasodine, which are known to diminish P-gp function [40]. Solamargine has been shown to decrease MDR1 mRNA expression [41], and reduce P-gp expression [40]. P-gp expression has been linked to the structural integrity of cells [42], suggesting that the loss of cellular morphology may contribute to the inhibition observed. Other steroidal alkaloids, such as tomatidine and cyclopamine, have also been found to inhibit P-gp [42]. Steroidal alkaloids may act as non-competitive inhibitors of P-gp. Active transport of compounds across the cellular membrane occurs at two distinct sites (H and R) in the transmembrane region [43]. Since steroidal alkaloids are small compounds, which possess a nitrogen atom and have a planar structure, they diffuse rapidly into cells. As such, compounds may interact easily with membrane carriers or transporters [43], decreasing activity.

Solamargine was shown to enhance the cytotoxic effect of doxorubicin in both SH-SY5Y and SK-Br3 cancerous cell lines. This potentiated response was not observed in non-cancerous cell lines, which suggests solamargine may be effective in improving treatment regimens for both neuroblastoma and certain breast cancers. As previously mentioned, in the present study P-gp inhibition only occurred at cytotoxic concentrations, thus enhancement of doxorubicin cytotoxic effects through P-gp inhibition seems improbable at the concentrations tested. The additive effects observed in the present study may indicate that the samples and doxorubicin target the similar pathways. Both doxorubicin and the samples have been proposed to mediate cell death via apoptosis. An antagonistic interaction would, such as that seen in the C2C12 cell line, suggests conflicting mechanisms on the same pathway [44]. Other species of *Solanum* have also been shown to provide conflicting combinational effects. For example, both synergistic and antagonistic interactions have been described for extracts of *S. nigrum* [45, 46]. The bioactive constituents are proposed to be steroidal alkaloids, such as solamargine [47], which is known to increase the cytotoxicity of anticancer drugs, such as cisplatin [48]. Chaconine and solanine [49], as well as other P-gp inhibitors [50, 51] display similar effects.

## Conclusions

The crude extract, as well as the aqueous alkaloid-enriched fraction, displayed noteworthy cytotoxicity and P-gp inhibition towards both cancerous and non-cancerous cell lines. Solamargine was found to be responsible for potent, non-selective cytotoxicity and P-gp inhibition. Solamargine and the aqueous alkaloid-enriched fraction enhanced doxorubicin’s cytotoxicity through additive effects in select cell lines, whereas having indifferent and antagonistic responses in others. P-gp inhibition only occurred at concentrations higher than those eliciting cytotoxicity, suggesting an alternative mechanism underlying its additive effect with doxorubicin. Due to the non-selective bioactivity, further mechanistic studies are required to address the preliminary results so that drug development viability can be assessed.

## Abbreviations

CAM: complementary and alternative medicines
COSY: correlation spectroscopy
dH_2_O: distilled water
DMEM: Dulbecco’s Modified Eagle’s Medium
DMSO: dimethyl sulfoxide
EMEM: Eagle’s Minimum Essential Medium
ERK1/2: Extracellular signal-regulated protein kinases 1 and 2
F-10/11: fraction 10/11
FIX: fractional inhibitory index
HMBC: heteronuclear multiple bond correlation
HPLC: high-performance liquid chromatography
HSQC: heteronuclear single quantum coherence
IC_50_: half-maximal inhibitory concentration
NC: negative control
NMR: nuclear magnetic resonance
PBS: phosphate-buffered saline
PC: positive control
P-gp: P-glycoprotein
RPMI: Roswell Park Memorial Institute
SRB: sulforhodamine B
TLC: thin-layer chromatography
UPLC: ultra-high performance liquid chromatography
VC: vehicle control
WHO: World Health Organization
